# Efficacy of auranofin as an inhibitor of desmoid progression

**DOI:** 10.1038/s41598-022-15756-9

**Published:** 2022-07-13

**Authors:** Kan Ito, Yoshihiro Nishida, Shunsuke Hamada, Koki Shimizu, Tomohisa Sakai, Bisei Ohkawara, Benjamin A. Alman, Atsushi Enomoto, Kunihiro Ikuta, Hiroshi Koike, Jiarui Zhang, Kinji Ohno, Shiro Imagama

**Affiliations:** 1 Department of Orthopedic Surgery, Nagoya University Graduate School of Medicine, 65 Tsurumai, Showa, Nagoya, Aichi 466-8550 Japan; 2 Department of Rehabilitation Medicine, Nagoya University Hospital, 65 Tsurumai, Showa, Nagoya, Aichi 466-8550 Japan; 3 Department of Orthopedic Surgery, Aichi Cancer Center Hospital, 1-1 Kanokoden, Chikusa, Nagoya, Aichi 464-0021 Japan; 4 Department of Orthopedic Surgery, Nagoya Memorial Hospital, 4-305 Hirabari, Tempaku, Nagoya, Aichi 468-8520 Japan; 5 Division of Neurogenetics, Center for Neurological Diseases and Cancer, Nagoya University Graduate School of Medicine, 65 Tsurumai, Showa, Nagoya, Aichi 466-8550 Japan; 6 Department of Orthopaedic Surgery, Duke University School of Medicine, Durham, NC 27710 USA; 7 Department of Pathology, Nagoya University Graduate School of Medicine, 65 Tsurumai, Showa, Nagoya, Aichi 466-8550 Japan

**Keywords:** Cancer, Oncology

## Abstract

Anticancer drugs and molecular targeted therapies are used for refractory desmoid-type fibromatosis (DF), but occasionally cause severe side effects. The purpose of this study was to identify an effective drug with fewer side effects against DF by drug repositioning, and evaluate its efficacy. FDA-approved drugs that inhibit the proliferation of DF cells harboring S45F mutations of *CTNNB1* were screened. An identified drug was subjected to the investigation of apoptotic effects on DF cells with analysis of Caspase 3/7 activity. Expression of β-catenin was evaluated with western blot analysis, and immunofluorescence staining. Effects of the identified drug on in vivo DF were analyzed using Apc1638N mice. Auranofin was identified as a drug that effectively inhibits the proliferation of DF cells. Auranofin did not affect Caspase 3/7 activity compared to control. The expression level of β-catenin protein was not changed regardless of auranofin concentration. Auranofin effectively inhibited the development of tumorous tissues by both oral and intraperitoneal administration, particularly in male mice. Auranofin, an anti-rheumatic drug, was identified to have repositioning effects on DF. Since auranofin has been used for many years as an FDA-approved drug, it could be a promising drug with fewer side effects for DF.

## Introduction

Desmoid-type fibromatosis (DF) is a (myo-) fibroblastic like cell proliferating tumor with aggressive local invasiveness, but without distant metastases. Previously, treatment of DF was primarily surgery, but due to the extremely high local recurrence rate, conservative treatment has lately been recommended worldwide^1,2^. Among conservative treatments, "wait & see", meaning only careful follow up, should be the first choice according to the recent consensus statement^2^. However, if the tumor size continues to increase or there is an exacerbation of symptoms that interfere with the activities of daily living (ADL) or quality of life (QOL), effective medical treatment is required. A previous report indicated that in over 40% of cases, drug or surgical treatment is selected because of tumor growth or decreased ADL and QOL due to pain^3^. Drugs with relatively fewer side effects, such as non-steroidal anti-inflammatory drugs (NSAIDs) and anti-hormonal agents, may have been selected and used, but there is little evidence of their efficacy due to the small number of cases and absence of controlled studies^4,5^. As chemotherapy, doxorubicin-based regimen^6,7^ or a low-dose combination of methotrexate (MTX) and vinblastine (VBL)^8,9^ has been reported as being effective. But with doxorubicin, there are concerns about cardiotoxic side effects, while with weekly MTX + VBL^8^ Grade 3–4 side effects have been reported in more than 10% of patients. Recently, the clinical results of biweekly administration of MTX + VBL have been reported, and the incidence of side effects has decreased significantly, but it takes time for the drug to take effect^10,11^. The effectiveness of targeted therapy including sorafenib and pazopanib has also been reported^9,12^. These drugs also have bothersome side effects and are expensive. In addition, previous reports have made little mention of when to stop such drug treatment or to what extent DF will re-grow after the drug is discontinued. Therefore, identification of drug(s) that control(s) the proliferation of DF cells with few side effects is a pressing need.

The purpose of this study is to identify an agent from among existing drugs that effectively suppresses DF cell proliferation using the drug repositioning method, clarify its underlying mechanism, and evaluate its efficacy in a mouse model spontaneously developing DF.

## Results

### Drug repositioning

**Figure 1:**
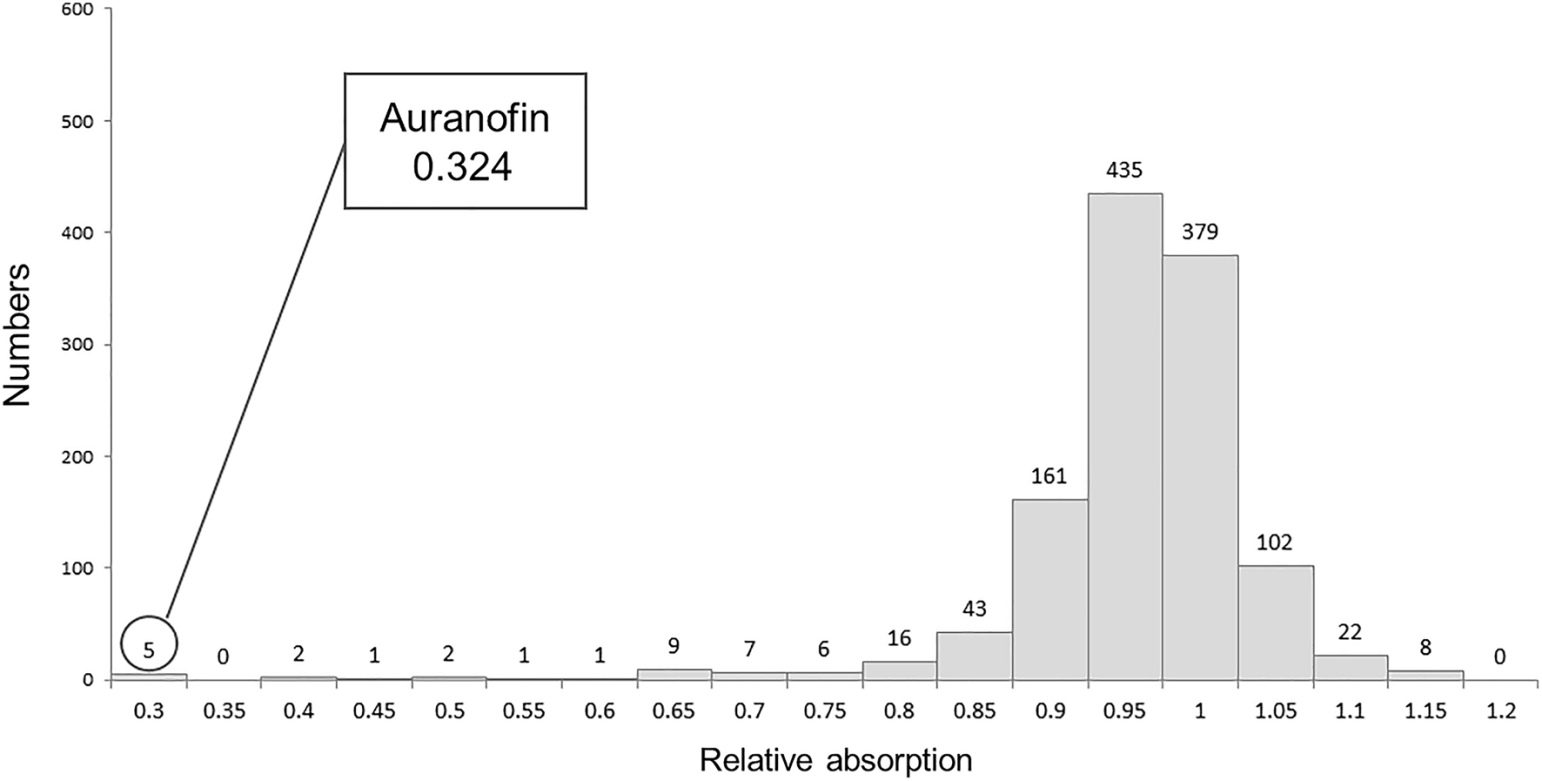
Screening for FDA-approved drugs. The S45F-mutated cells were cultured for 24 h in a 96 well culture plate of 5 × 10^3^ cells/well in the presence of 10 μM 1200 FDA-approved compounds. The cell proliferation was measured using the MTS assay, and the absorbance (at 490 nm) was measured using a microplate reader. The number of drugs is plotted for each growth inhibition rate of desmoid cells. If the relative absorption is less than 1, the growth inhibition will be stronger. Auranofin was extracted as an effective and safe drug, which had a proliferation rate of 0.324.

The results of drug screening are shown in Fig. 1. Ninety-three drugs suppressed desmoid cell proliferation by less than 0.9-fold. Of the 93 drugs, highly toxic and expensive drugs were excluded. Auranofin was extracted as an effective and safe drug, which had a proliferation rate of 0.324, approved by the Pharmaceuticals and Medical Devices Agency (PMDA, Japan). Among the drugs that had an inhibitory effect on DF cells, felodipine was selected as a control drug for subsequent experiments because it was a PMDA-approved and safe drug having the second-most inhibitory effect (proliferation rate is 0.869) after auranofin. The top 10 drugs that showed high inhibitory effects on DF cell proliferation are shown in Table 1.

**Table 1:** Drugs with low desmoid cell proliferation rate, their therapeutic groups and reference points.

Relative absorption	Chemical name	Therapeutic group	Reference
0.313	Terfenadine	Anti-histaminic	Discontinued due to cardiotoxicity
0.324	Auranofin	Anti-rheumatic	PMDA approved drug for rheumatoid arthritis
0.329	Alexidine dihydrochloride	Anti-biotic	Cleaning solution for contact lenses
0.344	Thimerosal	Anti-septic	Organic mercury compounds
0.346	Suloctidil	Vasodilator	Not available in Japan
0.42	Cantharidin	Dermal irritant	External medicine
0.442	Beta-Escin	Anti-inflammatory	Reagent
0.47	Ebselen	Anti-inflammatory	External medicine
0.501	Thonzonium bromide	Monocationic detergen	Ear drops
0.511	Mefloquine hydrochloride	Anti-malarial	PMDA approved drug for antiprotozoal

PMDA, Pharmaceuticals and Medical Devices Agency.

### MTS assays of DF cells under drug administration

**Figure 2:**
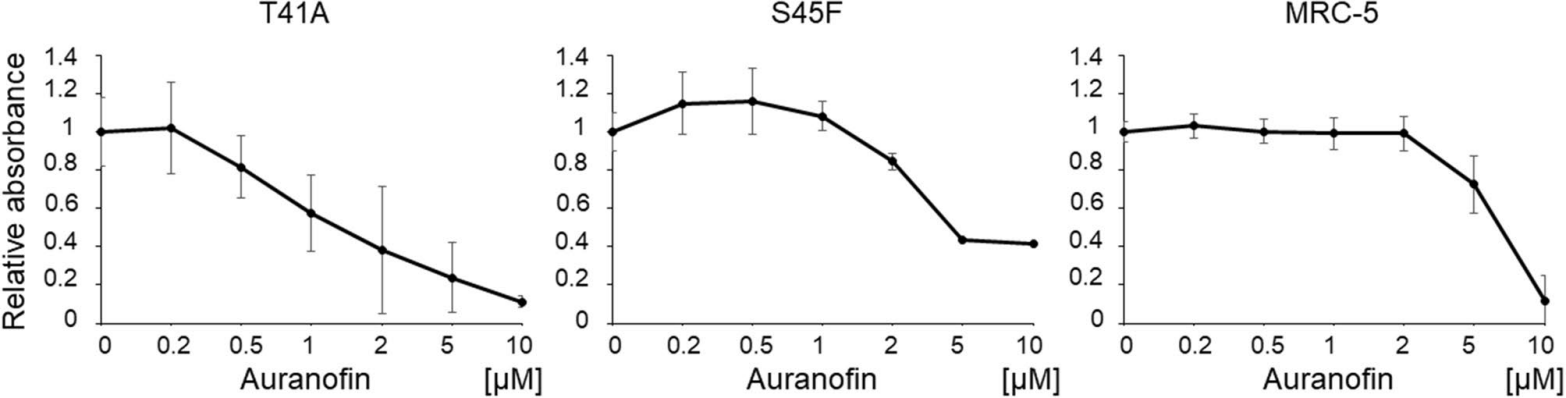
Effects of auranofin on DF cell proliferation. Inhibitory effects of auranofin on cell proliferation were evaluated with MTS assay. The DF cells were seeded on a 96-well plate (5 × 10^3^ cells/well) for 12 h. Thereafter, the effects of auranofin at each concentration (0.2 to 10 µM) on cell proliferation was measured using the MTS assay kit after 24 h.

The inhibitory effect of auranofin on the proliferation of DF cells was evaluated not only for cells with S45F mutation, which was used in drug screening, but also those with T41A mutation, and MRC-5 cells as a control fibroblast. Proliferation of S45F-mutated cells was significantly inhibited by 15.5% at 2 μM and 56.4% at 5 μM compared to 0 μM of auranofin (*P* = 0.004 and *P* = 0.002, respectively), and that of T41A was also significantly inhibited by 61.9% at 2 μM and 76.3% at 5 μM (*P* < 0.001 for each). Proliferation of MRC-5 cells was not inhibited up to 2 μM, but was by 27.4% at 5 μM (Fig. 2). MTS assays were also performed under felodipine treatment, however, felodipine did not inhibit proliferation of cells with T41A mutation compared to MRC-5 cells (Supplementary Figure S1 online). In addition, MTS assays were performed in T41A-mutated cells every 6 h up to 24 h after auranofin administration, with little change from 6 to 24 h (Supplementary Figure S2 online).

### Apoptosis and reactive oxygen species assay

**Figure 3:**
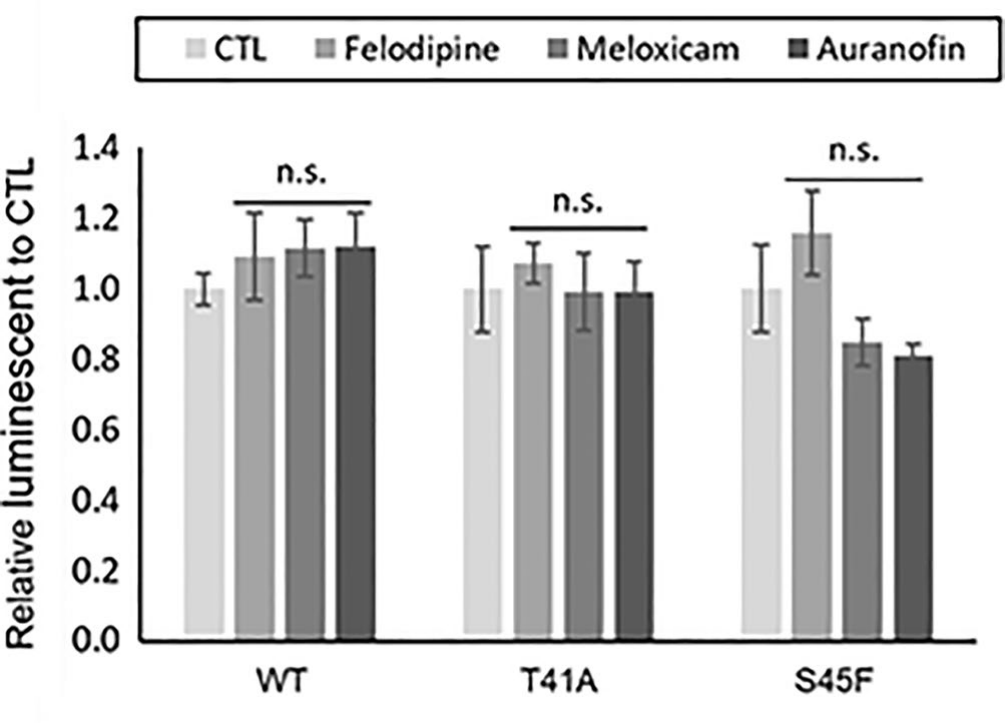
Apoptosis assay for DF cells under drug administration. The DF cells (WT, T41A, and S45F) were seeded on a 96-well plate (5 × 10^3^ cells/well) for 12 h. Thereafter, the effects of auranofin, felodipine, and meloxicam on the activity of Caspase 3/7 was measured using a Caspase assay kit). CTL: control, n.s.: not significant.

Auranofin, felodipine, and meloxicam did not show any significant increase in Caspase 3/7 activity as compared to the control. The results were similar for all DF cells of wild type (WT), T41A and S45F. (Fig. 3). The reactive oxygen species (ROS) transition of T41A-mutated cells treated with auranofin was observed, but there were no significant changes (Supplementary Figure S3 online).

### Effects of auranofin on expression of β-catenin and downstream genes of Wnt/β-catenin signaling pathway

**Figure 4:**
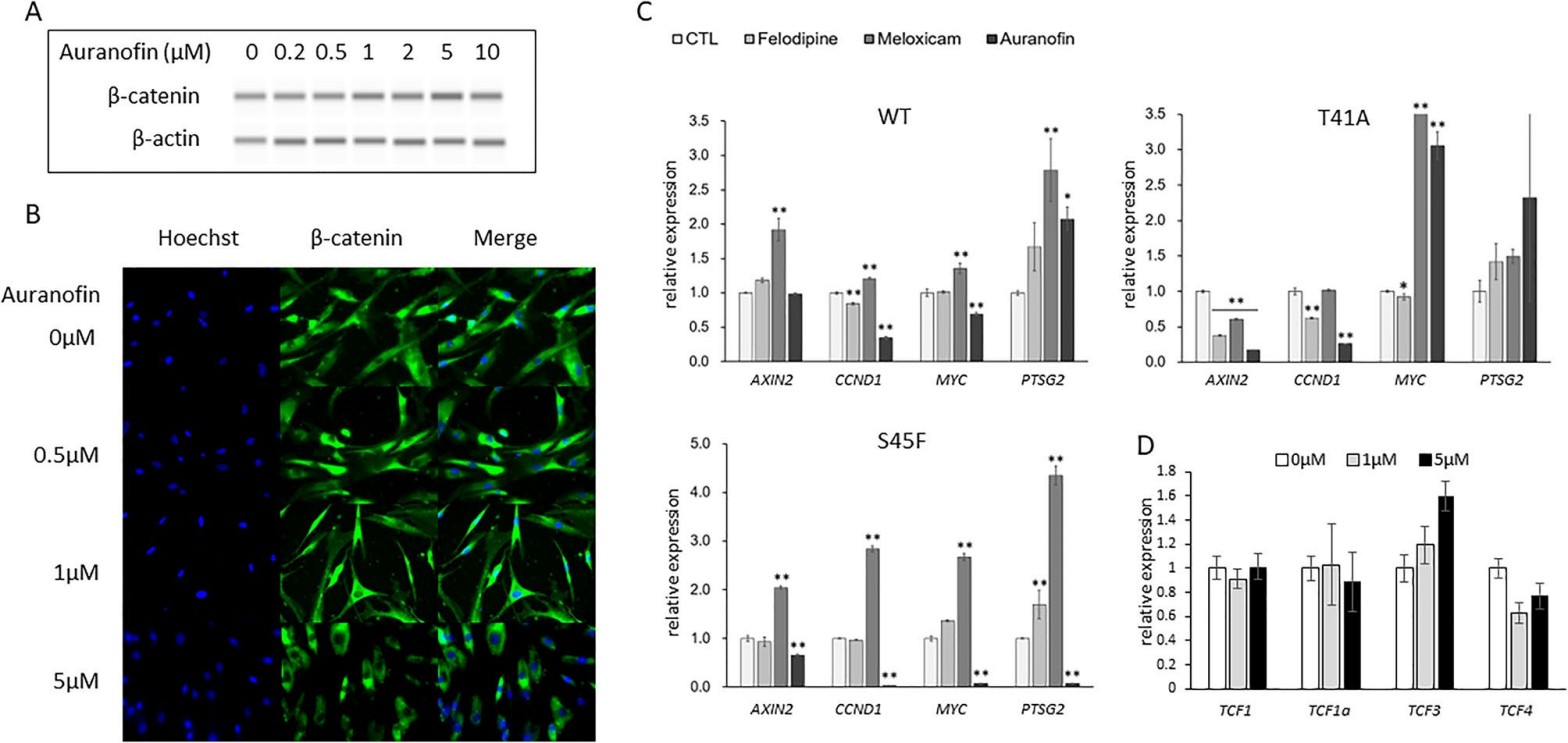
Effects of auranofin on expression of β-catenin and downstream genes of Wnt/β-catenin signaling pathway. (**A**) Effects of auranofin (0–10 µM) on the expression of β-catenin protein. The T41A-mutated cells were seeded on a 6-well plate (1 × 10^5^ cells per well) for 12 h followed by auranofin (0–10 µM) treatment for 24 h. These cells were subjected to western blotting using Simple Western for β-catenin and β-actin. (**B**) Fluorescent immunostaining for β-catenin. The T41A-mutated cells were seeded onto chamber slides (1.0 × 10^5^ cells/ml) for 12 h followed by auranofin treatment (0–5 µM) for 24 h. Cells were fluorescently visualized using mouse anti-β-catenin antibody. (**C**) Expression levels of mRNA, *AXIN2, CCND1, MYC, PTGS2*. The DF cells (WT, T41A and S45F: 1 × 10^4^ cells per well) were seeded in a 96-well plate for 12 h. Subsequently the cells were cultured in a medium containing felodipine, meloxicam, and auranofin (5 μM) for 6 h, and subjected to RT-PCR. CTL: control. (**D**) Effects of auranofin (0–5 µM) on the mRNA expression of four TCFs. The T41A cells (1 × 10^4^ cells per well) were seeded in a 96-well plate for 12 h. Subsequently the cells were cultured in a medium containing auranofin (0–5 µM) for 6 h, and subjected to RT-PCR. *P < 0.05, **P < 0.01.

With T41A-mutated cells, the expression level of β-catenin was not affected by the administration of various concentrations of auranofin as compared with control in Simple Western assays (Fig. 4A) (see Supplementary Figure S4 online).

We observed the distribution of β-catenin in T41A-mutated cells under 0-5 μM auranofin treatment using fluorescence immunostaining. The distribution of β-catenin in the nucleus and cytoplasm did not appear to change according to the auranofin concentration. However, the cell shape was slightly rounded by the administration of 5 μM auranofin (Fig. 4B).

The administration of auranofin, felodipine, and meloxicam resulted in various changes in mRNA expression of *AXIN2* (*AXIN-2*), *CCND1* (*CYCLIN D1*), *MYC* (*c-MYC*), and *PTGS2* (*COX-2*). Notably, auranofin suppressed *CCND1* in all of WT, T41A, and S45F cells. In the S45F-mutated cells, auranofin suppressed the expression of all four genes (Fig. 4C). Analysis of protein expression levels of these four genes in T41A-mutated cells with auranofin using Simple Western assay showed reduced expression of all proteins (Supplementary Table S1 online).

The mRNA expression levels of *TCF1* / *TCF1a* / *TCF3* / *TCF4* on treatment with auranofin were measured. We found that *TCF3* increased in a concentration-dependent manner with auranofin (Fig. 4D).

### Effects on a spontaneous DF developing mouse model

**Figure 5:**
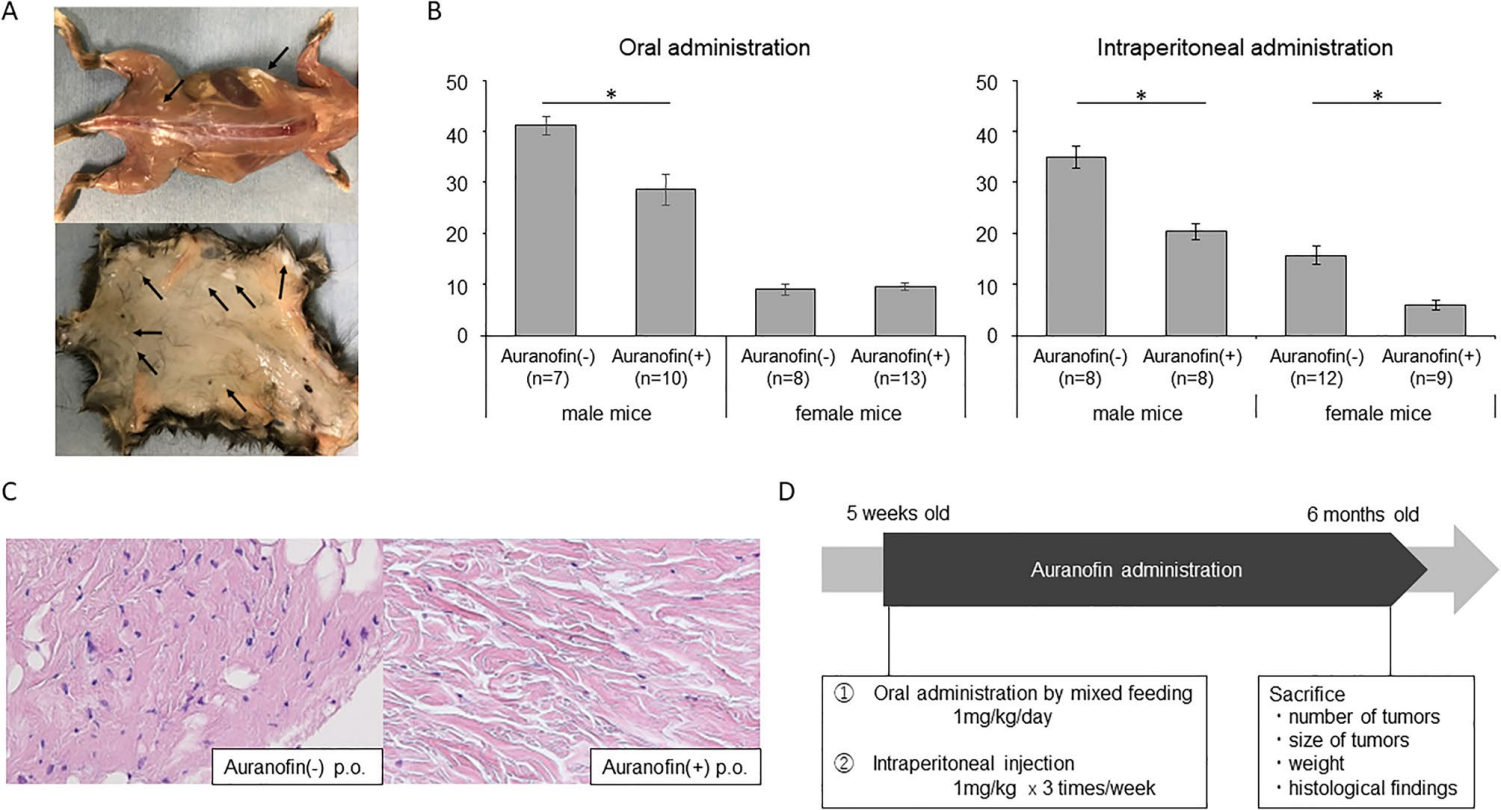
Schedule of auranofin administration for Apc1638N mice and effects of auranofin in in vivo DF development model mice. (**A**) Representative findings of tumor development in a Apc1638N mouse (6 months of age) (black arrows: tumorous tissues). Small white tumorous tissues of a few millimeters in size occurred subcutaneously and on the fascia. (**B**) The number of tumors that developed in Apc1638N (6 months of age) with or without treatment of auranofin by oral or intraperitoneal administration. Oral dose for mice was set at 1.0 mg/kg/day and the intraperitoneal dose was set as 1 mg/kg three times a week. The average number of tumors developed with oral administration of auranofin (10 male mice and 13 female mice) and control (7 male mice and 8 female mice), ant that with intraperitoneal administration of auranofin (8 male mice and 9 female mice) and control (8 male mice and 12 female mice) were plotted. *P < 0.01. (**C**) Pathological findings of the tumors developed in mice with or without treatment of auranofin by oral administration (6 months of age) (H&E, × 200). p.o.: per OS. (**D**) Experimental procedure in vivo. Auranofin was administered from 5 weeks to 6 months of age. Two separate experiments were performed based on the administration route, oral and intraperitoneal. Mice were sacrificed at 6 months of age, and the number of tumors, tumor size, tumor weight were calculated. Tumorous tissues were also subjected to histological examination.

Tumors had developed in the subcutaneous tissue, fascia, and muscle of Apc1638N mice by the time of sacrifice (6 months of age) (Fig. 5A). First, regarding the oral administration experiment, the average number of tumors in male mice treated with auranofin was 41 ± 4.6 in the control group (n = 7) and 29 ± 9.0 in auranofin group (n = 10), showing a statistically significant decrease in the number of tumors in auranofin group (*P* = 0.007) (Fig. 5B). On the other hand, the average number of tumors in female mice was 9 ± 2.9 in control group (n = 8) and 10 ± 2.5 in auranofin group (n = 13), with no significant difference (*P* = 0.86). There was no significant difference in the bodyweight of the mice between the control and auranofin groups (*P* = 0.36 for males and *P* = 0.41 for females).

In the experiments with intraperitoneal administration, the average number of tumors in male mice was 35 ± 5.6 in control group (n = 8) and 20 ± 4.0 in auranofin group (n = 8), indicating a statistically significant decrease in the number of tumors in auranofin group (*P* < 0.001). In female mice, the average number of tumors in control group was 16 ± 6.4 (n = 12) and 6 ± 2.5 in auranofin group (n = 9), showing a statistically significant decrease in the number of tumors in auranofin group (*P* < 0.001). There was no significant difference in the bodyweight of the mice between the control and auranofin groups (*P* = 0.44 for males and *P* = 1.0 for females).

The average size of the tumors that developed in all male mice was 1.9 mm and that in females was 1.6 mm. There was no significant difference in tumor size between control and auranofin groups for either oral or intraperitoneal administration (for oral administration in male mice *P* = 0.14, in female mice *P* = 0.38; for intraperitoneal administration in male mice *P* = 0.32, in female mice *P* = 0.24).

Histological evaluation showed less cellularity in the tumorous tissues in the auranofin-treated group as compared with control (Fig. 5C). There seemed to be no difference in the nuclear staining of β-catenin between the auranofin-treated and non-treated groups (see Supplementary Fig. S5 online).

## Discussion

When the "wait and see policy", which is the initial treatment modality for DF, fails, the use of effective medication is recommended^2^ in locations other than the abdominal wall. This means Dox-based^6,7^, low-dose chemotherapy with MTX + VBL^8,9,11,13^, and molecular-targeted therapy with sorafenib^12^ or pazopanib^9^. The therapeutic effects of these drugs have been proven, but severe side effects have also been reported. The development of a promising drug treatment for DF with fewer side effects is awaited. In the present study, by a drug repositioning method auranofin was identified as a possible agent.

The pathogenesis of DF is mainly divided into two types: a germline mutation in the *APC* and a sporadic mutation in exon 3 of *CTNNB1* gene. The treatment algorithm has been reported to be quite similar for both types^2^. However, several studies have shown that mutation types of *CTNNB1* affect various treatment outcomes. Previous studies indicated that patients with S45F mutation have a high recurrence rate after surgical treatment^14,15^. Recently, some studies have analyzed whether mutation types affect the outcome of medical treatment. One study showed that DF patients with S45F mutation had a poor outcome with meloxicam, which is a selective COX-2 inhibitor NSAID^16^. Based on these previous reports, the S45F type is considered potentially to have an aggressive character in DF. For that reason, DF cells with the S45F mutation were used for drug screening in the present study.

Hotspot sites have been reported in the *CTNNB1* mutation, which are thought to underlie approximately 85% of the cases of DF^17^. A review of the *CTNNB1* mutation type in DF summarized eight analyzes, with T41A being the most common, followed by S45F, and WT^18^. Based on these reports, it seemed to make sense to perform the in vitro evaluation on these three types of cell in the present study.

Auranofin is an alkylphosphine gold coordination complex that has long been used clinically in the treatment of rheumatoid arthritis, and has shown stable safety in humans^19,20^. Several studies have been conducted to determine whether auranofin can be used as a therapeutic agent for malignant tumors, neurodegenerative diseases such as Alzheimer's disease and Parkinson's disease, parasitic infections, and bacterial infections^21^. One mechanism of auranofin activity has been thought to be mediated by the inhibition of reduction/oxidation (redox) enzymes such as thioredoxin reductase (TrxR) that are essential for maintaining intracellular levels of ROS^21^. By inhibiting these enzymes, auranofin increases ROS and induces apoptosis and further induces DNA damage by causing mitochondrial dysfunction^22–24^. Furthermore, referring to the relationship between ROS and the Wnt/β-catenin signaling, it has been reported that Ape1 and Nrf2 degrade or alter the localization of β-catenin via ROS^25,26^. In the present study, no obvious induction of apoptosis or increase in ROS was observed, suggesting that auranofin may inhibit cell proliferation without relating to these pathways.

To our knowledge no reports have focused on the effects of auranofin on Wnt/β-catenin signaling. The results of the present study indicated that auranofin treatment partially suppressed the expression of genes downstream of the Wnt/β-catenin signal, suggesting that auranofin may act on this signaling pathway. In addition, when the intracellular localization of β-catenin was examined, changes in the distribution after drug administration were unclear with western blot and fluorescent immunostaining in vitro. These results suggest that auranofin acts after translocation of β-catenin into the nucleus, that is, in the process of binding to T-cell factor (TCF), and/or transcription of downstream genes. Therefore, we assayed the mRNA expression of *TCF1* / *TCF1a* / *TCF3* / *TCF4* and found an increase in *TCF3.* Since TCF3 is generally known to suppress Wnt target gene expression^27,28^, the results of the present study may suggest that auranofin inhibits tumor growth through increasing TCF3. On the other hand, the possibility that some kind of gold transporter might play a role in the weak effect of auranofin in the S45F mutant cells cannot be ruled out. Further experiments are needed to elucidate the detailed mechanism of the action.

Two mouse models in which DF develops have been reported, a *Ctnnb1* gene mutation model in which Ng2/Cspg4-CreER mouse is crossed with a Ctnnb1^lox(exon3)^ mouse^29^, and an Apc1638N mouse model (*Apc* gene mutant model)^30^. To determine the more appropriate mouse model for use in the present experiment, a preliminary experiment was conducted to determine whether both models developed tumors. Since the number of tumors was low, and there was no reproducibility in the former model, the latter model, in which tumors sometimes occur, was used in the present study. APC forms a complex with glycogen synthase kinase 3 beta (GSK3B) and contributes to the stabilization of β-catenin. However, when a mutation occurs in *APC*, this complex inhibits phosphorylation of β-catenin, and β-catenin is no longer degraded and accumulates in cells. The translocation of accumulated β-catenin into the nucleus has been thought to result in the onset of desmoid^31–33^. Therefore, it seems to be appropriate to use *Apc* mutant mice to evaluate drug efficacy against DF although *APC* mutations have been reported to account for DF onset in 15% of cases. In the present study, auranofin was administered to these mice at a dose calculated to be equivalent to that used in humans as an anti-rheumatic drug suggesting clinical applicability.

The results of the preliminary experiments showed that the number of tumors of DF increased sharply after 6 weeks of age, which is in consistent with a report on Apc1638N mice by Smits et al.^30^. Regarding the number of DF tumors that develop, there is no major difference between the reports of Smits and the present study (Smits: 46 for male mice, 16 for females at the age of 6 months, present study: 41 for males, 9 for females). Similar results were also seen regarding the more frequent occurrence in male mice.

The results of the present in vivo study revealed that auranofin treatment suppressed the number of tumors but not reduce the tumor size. Due to the small size of the tumor that develops in this mouse model, it may be difficult to use this model for the purpose of assessing tumor size and growth. Although no size difference was observed between the auranofin-treated and control groups, the histological findings indicated that cellular density was lower in auranofin group than in control group, indicating the suppression of tumor aggressiveness by auranofin.

To apply therapeutic treatments for human, there is some advantages but it also remains unknown things for auranofin. First, in the mouse model used, the number of tumors that developed in males was high. On the other hand, in humans, DF occurs more frequently in women. Although the mechanism underlying the higher frequency of DF occurrence in male mice is unknown, intraperitoneal administration of auranofin significantly suppressed the onset of DF not only in male mice but also in female ones, suggesting that a tumor-suppressing effect can be expected in both genders. Second, since human desmoid shows mutations more frequently in *CTNNB1* than *APC,* there is concern about the effectiveness of auranofin for patients with *CTNNB1* mutation. There is a consensus statement that there is no need to change drug treatment options between desmoid that develops with an *APC* mutation in the germ line (Gardner's syndrome) and desmoid that has a *CTNNB1* mutation in sporadic cases^2^ based on the findings that both tumor types respond to drug treatment similarly^11^. Third, the time required for the effect of auranofin to appear has not been determined yet. For human use, it is necessary to clarify the time until the therapeutic effect of the drug appears.

Finally, the present study identified and confirmed auranofin as a potentially effective drug for DF by drug repositioning method. Auranofin is a drug that has been used for RA patients for many years and is inexpensive with relatively low toxicity. Although there are anticancer drugs and molecular-targeted drugs that have been shown to be effective against DF, auranofin is an FDA-approved drug and considered to be an easy-to-use drug for DF, which is an intermediate tumor. In order to obtain a proof of concept for clinical use, an investigator-initiated clinical trial is now being planned.

## Methods

### DF cell culture

Informed consent was obtained from all patients. All research was performed in accordance with relevant guidelines/regulations, and the Declaration of Helsinki. Tumor tissues pathologically diagnosed as DF were obtained during incisional biopsy or excision. DF cells with three different mutation types on the gene, human *CTNNB1* encoding β-catenin, were prepared: WT, T41A- and S45F. Tumorous tissues were cut into small fragments, and lysed by incubation at 37 °C for 3 h in Dulbecco's modified Eagle's medium (DMEM) supplemented with 0.2 mg/mL proteinase. Cells separated from lysates were mono-layer cultured in DMEM containing 10% fetal bovine serum (FBS) supplemented with 100 U/mL penicillin and 100 mg/mL streptomycin at 37 °C with 5% CO_2_. The culture medium was changed every 3–4 days, and cells were passaged by trypsinization before reaching confluence. Since beyond 20 passages, DF cells show senescence and are not suitable for experimentation, DF cells of passages 5–15 were used for the experiments.

### Screening of FDA-approved compounds using DF cells with S45F mutation

The S45F-mutated cells were cultured for 24 h in a 96 well culture plate of 5 × 10^3^ cells/well in the presence of 10 μM 1200 FDA-approved compounds (Prestwick Chemical Libraries, Illkirch-Graffenstaden, France). The cell proliferation was measured using the MTS assay (CellTiter 96 AQueous Assay, Promega, Madison, WI) according to the manufacturer’s instructions, and the absorbance (at 490 nm) was measured using a microplate reader, Rainbow RC (Tecan, Männedorf, Switzerland).

### Cell proliferation assay

The identified drug was auranofin, an anti-rheumatic drug. Auranofin was subsequently used for evaluation of various inhibitory effects on DF. The DF cells were seeded on a 96-well plate (5 × 10^3^ cells/well) in DMEM containing 10% FBS for 12 h. Thereafter, the medium was replaced with a medium containing 10% FBS and 0.68% dimethyl sulfoxide (DMSO) or auranofin (dissolved in DMSO) at each concentration of 0.2 to 10 µM. The cell proliferation after 24 h was measured using the MTS assay kit, and the absorbance was measured using Rainbow RC. MRC-5 cells, fibroblasts originally developed from the lung tissue of male fetus (provided by the Riken BioResource Center, Tsukuba, through the National BioResource Project of the MEXT/AMED, Japan) were used as control cells. For felodipine, which was identified as a control drug, the MTS assay was performed in the same manner.

### Apoptosis and ROS assay

The DF cells were seeded on a 96-well plate (5 × 10^3^ cells/well) for 12 h in DMEM containing 10% FBS. Thereafter, the medium was replaced with a medium containing 10% FBS with DMSO or 5 µM of each drug (dissolved in DMSO), and the plate was incubated for 24 h. The drugs used in the analysis are: auranofin, felodipine that was the second most effective drug after auranofin, and meloxicam, which has been used for DF in our institution as an NSAID^4^. The activity of Caspase 3/7, which is an indicator of the apoptosis-inducing ability, was measured using a Caspase assay kit (Caspase-Glo 3/7 Assay, Promega) at the 24 h time point, and the luminous intensity was measured using a microplate reader, PowerScan4 (DS Pharma Biomedical, Osaka, Japan).

Similarly, in the ROS assay, 2.5 × 10^4^ T41A-mutated cells per well were seeded and incubated for 12 h, and then treated with a medium containing 10% FBS with DMSO or 1–10 µM of auranofin (dissolved in DMSO) for 24 h. The ROS Detection Assay Kit (BioVision, Waltham, MA) was used as described in its instructions, and fluorescence intensity was measured in the microplate reader.

### Simple Western assays

The T41A-mutated cells were seeded on a 6-well plate (1 × 10^5^ cells per well) in DMEM containing 10% FBS for 12 h. Thereafter, the medium was replaced with DMEM containing 10% FBS and DMSO with or without auranofin (dissolved in DMSO) at 0–10 µM concentration, and the plate was incubated for 24 h. These cells were collected and lysed on ice with radioimmunoprecipitation (RIPA) lysis buffer, and then centrifuged (1800 rpm, 10 min, 4 °C). Protein concentrations were determined using Pierce BCA Protein Assay Kit (Thermo Fisher Scientific, Waltham, MA). Western blotting was performed using a Jess Simple Western system, and an automated capillary-based size sorting system (ProteinSimple, San Jose, CA). The expression level of β-catenin in these DF cells was evaluated according to the manufacturer’s standard method for 12–230 kDa Jess separation module (SM-W004). Briefly, the extracted samples were mixed with 0.1X Sample buffer and Fluorescent 5X Master mix (ProteinSimple) to achieve a final concentration of 1 μg/μL in the presence of fluorescent molecular weight markers and 400 mM dithiothreitol (ProteinSimple). This preparation was denatured at 95 °C for 5 min. Ladder (12–230-kDa PS-ST01EZ), and proteins extracted from DF cells were separated in capillaries as they migrated through a separation matrix. The antibodies of β-actin (#4970, 1:50 dilution, Cell Signaling Technology, Danvers, MA), β-catenin (#9562, 1:50 dilution, Cell Signaling Technology), AXIN2 (#20540–1-AP, 1:50 dilution, Proteintech, Rosemont, IL), CCND1 (ab16663, 1:10 dilution, Abcam, Cambridge, UK), MYC (C3956, 1:25 dilution, Sigma-Aldrich, St. Louis, MO), and PTGS2 (#12375–1-AP, 1:25 dilution, Proteintech) were used as the primary antibodies. The HRP-conjugated anti-rabbit secondary antibody was applied according to the instructions of Simple Western kit (ProteinSimple). Chemiluminescence reactions with antibodies were measured and their digital blot images were constructed by the Compass software (ProteinSimple).

### Fluorescent immunostaining

The T41A-mutated cells in DMEM containing 10% FBS were seeded onto chamber slides (FALCON, Big Flats, NY) at 1.0 × 10^5^ cells/ml and incubated for 12 h. The culture medium was replaced with 10% FBS-containing DMEM containing auranofin (dissolved in DMSO) at each 0–5 µM concentration and cultured for 24 h, followed by fixation with 4% paraformaldehyde for 2 h at room temperature. Cells were permeabilized in phosphate-buffered saline (PBS) containing 0.2% Triton X-100 by incubation at room temperature for 10 min. Preparations were incubated in blocking buffer containing 10% goat serum for 60 min at room temperature and incubated with mouse anti-β-catenin antibody (1:500 dilution, BD Biosciences, Franklin Lakes, NJ) at 4 °C overnight. Next, they were incubated with goat anti-mouse fluorescein isothiocyanate (FITC) secondary antibody (1:100 dilution, Proteintech) in a dark environment for 1 h at room temperature. Hoechst 33342 (5 μL/mL, Thermo Fisher Scientific) was added to them and incubated for 10 min at room temperature in a dark environment.

### mRNA expression analysis: reverse transcriptase-polymerase chain reaction (RT-PCR)

To evaluate the influence of each drug on the Wnt/β-catenin signaling pathway, the mRNA expression levels of downstream genes *AXIN2, CCND1, MYC,* and *PTGS2*, ^34^ were measured. The DF cells (1 × 10^4^ cells per well) with WT, T41A and S45F mutation were seeded in a 96-well plate in DMEM containing 10% FBS for 12 h. Subsequently the cells were cultured in a medium containing 5 μM of each drug for 6 h, and total cellular RNA was extracted using RNeasy Mini Kit (Qiagen, Hilden, Germany) according to the instructions. The cDNA obtained by the RT was subjected to a real-time PCR reaction using LightCycler (Roche Diagnostics, Mannheim, Germany). In addition, for only T41A-mutated cells, mRNA expression of *TCF1*, *TCF1a*, *TCF3*, and *TCF4* was evaluated in a similar manner. The expression level of mRNA of each gene was evaluated as a relative value, with reference to the expression level of *GAPDH* used as an internal control. The primers of *AXIN2*, *CCND1*, *MYC, PTGS2, TCF1*, *TCF1a*, *TCF3*, *TCF4* and *GAPDH* are listed in Supplementary Table S2 online.

### Effects in vivo on mice spontaneously developing desmoid

All animal procedures for experiments were approved by the Animal Care and Use Committee of Nagoya University (license number: 28106), and carried out according to the National Institutes of Health's Guide to the Management and Use of Laboratory Animals. This research was conducted in compliance with ARRIVE (Animal Research: Reporting of In Vivo Experiments) guidelines and relevant regulations. Apc1638N mice have been reported as a spontaneous DF onset model^30^. In the same report, desmoid-like tumors were reported to develop frequently after approximately 6 weeks of age. Therefore, auranofin administration was started at 5 weeks of age in the present study. Mice were fed a diet with or without auranofin. As another experiment to change the route of administration, intraperitoneal administration and a control group were set up, and administration was started at 5 weeks of age. In terms of dose of the drug, auranofin normally used in humans is 6 mg/day, which is calculated to be 0.1 mg/kg/day assuming a bodyweight of 60 kg. Since the human equivalent dose for mice is 12.3^35^, oral dose for mice should be set at 1.23 mg/kg/day, but was set at 1.0 mg/kg/day in the present study. In terms of intraperitoneal dose, the bioavailability of auranofin in rats has been reported to be 20–30% with oral administration^36^ compared with intraperitoneal administration. Oral dose (1.23 mg/kg X 7 days) appears to be the same as the intraperitoneal dose (3 mg/kg/week, bioavailability 20–30%). Therefore, the intraperitoneal dose was set as 1 mg/kg three times a week. Mice were sacrificed with the cervical dislocation procedure by a proficient person while sedated with 4% isoflurane. The number of tumors developed, tumor size, body weight, and histological features at 6 months after birth were compared between the auranofin and control groups, in male and female mice, respectively. A scheme of this experimental protocol is shown in Fig. 5D. In the oral experiment, 23 cases of auranofin group (10 male mice and 13 female mice) and 15 cases of control group (7 male mice and 8 female mice) were analyzed. In the intraperitoneal experiment, 17 cases of auranofin group (8 male mice and 9 female mice) and 20 cases of control group (8 male mice and 12 female mice) were analyzed. Initially, we estimated at least 8 mice were required in each group, and assigned more mice to each. Some mice dying of unknown cause were present in each group, resulting in differences in the number of mice per group.

All tumor samples detected were obtained by resection, fixed in 10% formaldehyde, and embedded in paraffin. Sections were cut to a thickness of 4 μm and subjected to hematoxylin–eosin staining and immunostaining using β-catenin antibody. According to a previous report^37^, the slides were treated overnight at 4 °C with anti-β-catenin antibody (ab2365, Abcam), counterstained with hematoxylin, dehydrated, and mounted.

### Statistical analysis

Mann–Whitney U test was used to compare the results of the cell proliferation assay, apoptosis assay, and RT-PCR. Differences in tumors developing in mice treated and untreated with auranofin were also analyzed by Mann–Whitney U test. Statistical analyses were conducted using SPSS 28 (IBM Corporation, Armonk, NY). In all analyses, *P*-values < 0.05 were considered statistically significant.

### Institutional review board statement

This study was approved by Institutional Review Board of Nagoya University Hospital (approved number: 2014-0280).

### Informed consent statement

Informed consent was obtained from the patients with DF involved in the study.

## Supplementary Information

Supplementary Information.

## Data Availability

The datasets generated and/or analyzed during the current study are available from the corresponding author on reasonable request.
