# Supplementary material for: Efficacy of auranofin as an inhibitor of desmoid progression
Source: Sci Rep. 2022 Jul 13;12:11918. doi: 10.1038/s41598-022-15756-9 (PMC9279441; doi:10.1038/s41598-022-15756-9)
Supplement: Supplementary file 1 — Supplementary Information. [file 41598_2022_15756_MOESM1_ESM.pdf]

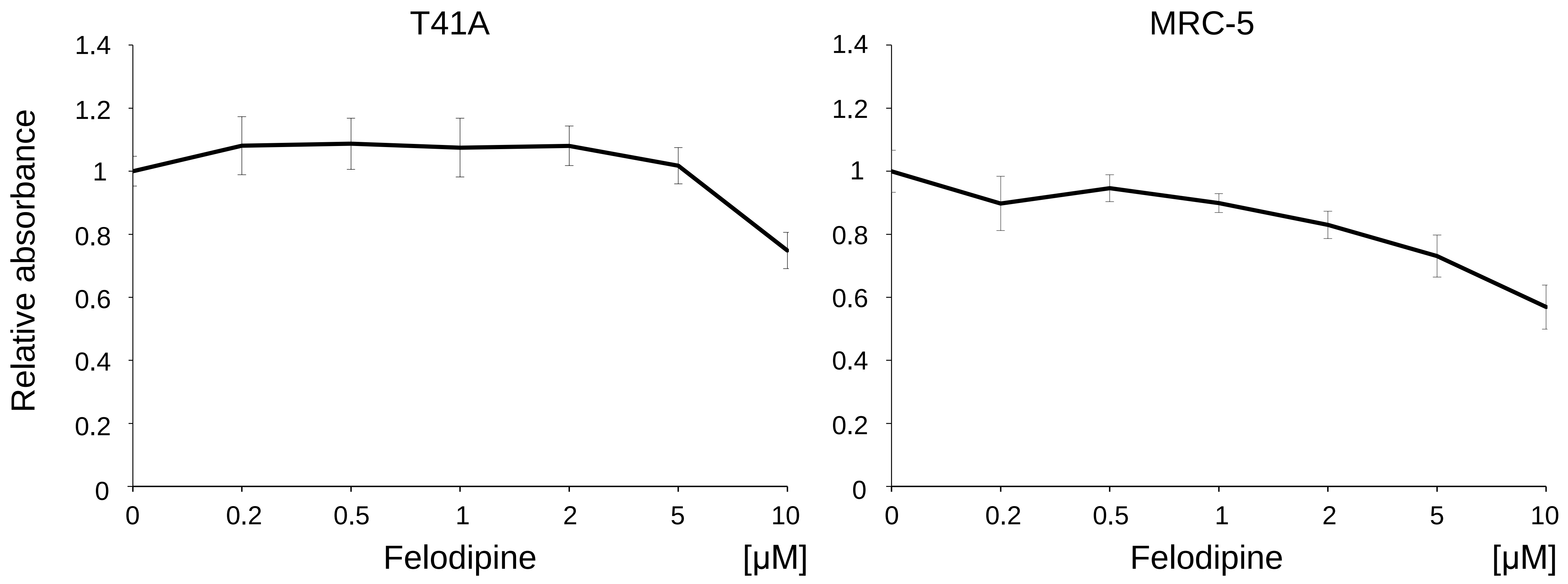

Suppl. Fig. S1. MTS assay with Felodipine.

The DF cells were seeded on a 96-well plate (5×10^3 cells/well) for 12 hours. Thereafter, the effects of felodipine at each concentration (0.2 to 10μM) on cell proliferation was measured using the MTS assay kit after 24 hours.

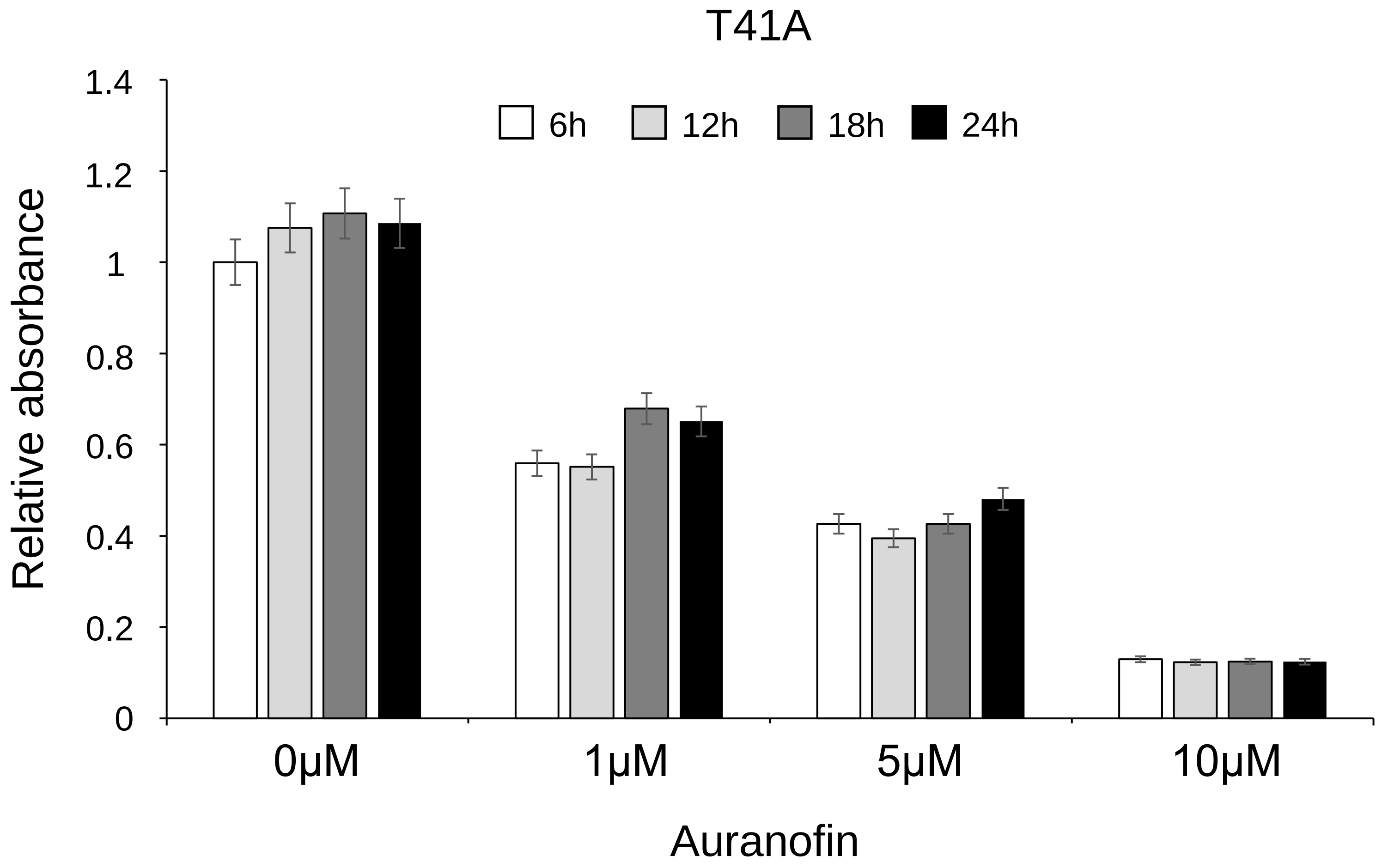

Suppl. Fig. S2. Time course of the MTS assay under auranofin administration.

The DF cells were seeded on a 96-well plate (5×10^3 cells/well) for 12 hours. Thereafter, the effects of auranofin at each concentration (1 to 10μM) on cell proliferation was measured using the MTS assay kit 6, 12, 18, and 24 hours after drug administration.

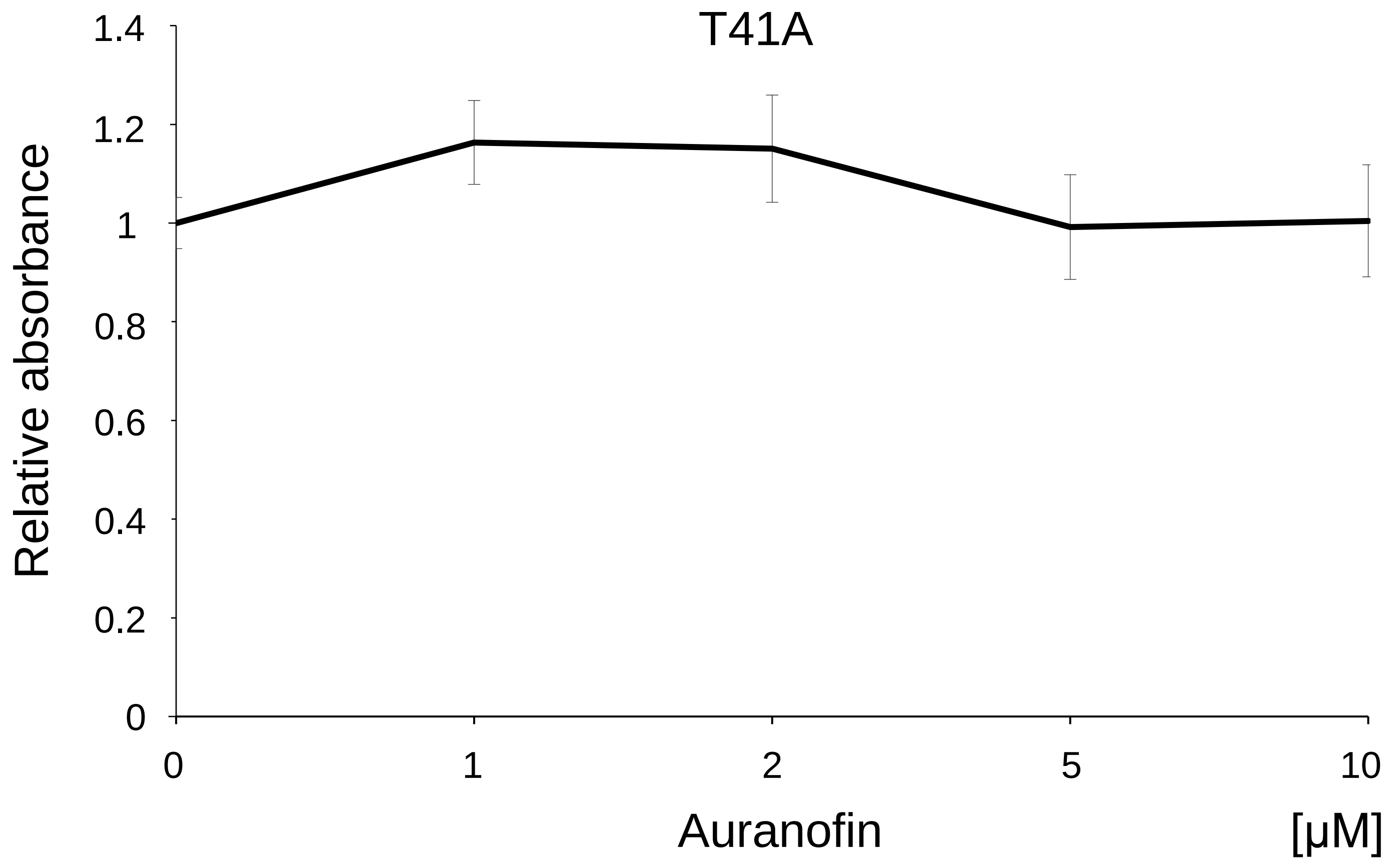

Suppl. Fig. S3. Results of ROS assay in T41A-mutated cells treated with auranofin.
T41A-mutated cells (2.5×10^4 cells/well) were seeded and incubated for 12 hours, and treated with 1-10 µM of auranofin for 24 hours and subjected to the ROS assay.

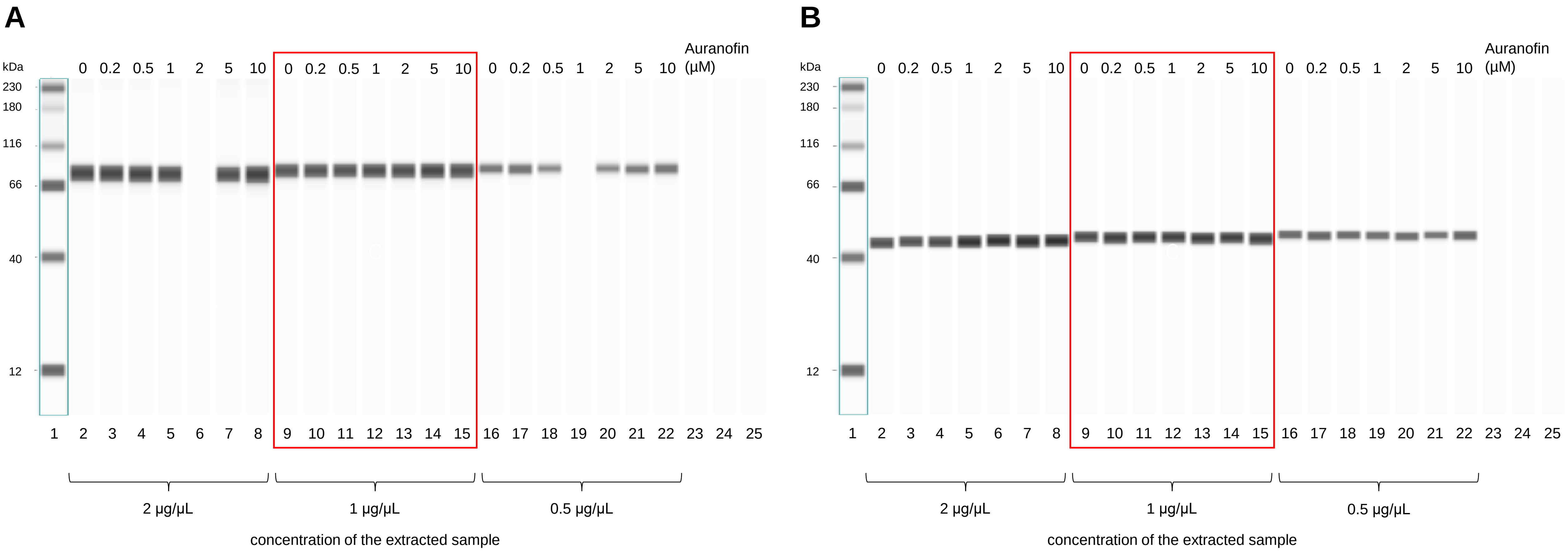

Suppl. Fig. S4. Full-length blots of Simple Western assay.
The results of Simple Western assay when β-catenin antibody (A) and β-actin antibody (B) were used as primary antibodies are shown. The result when the final concentration of the extracted sample was 1 μg/μL was adopted (in the red box).

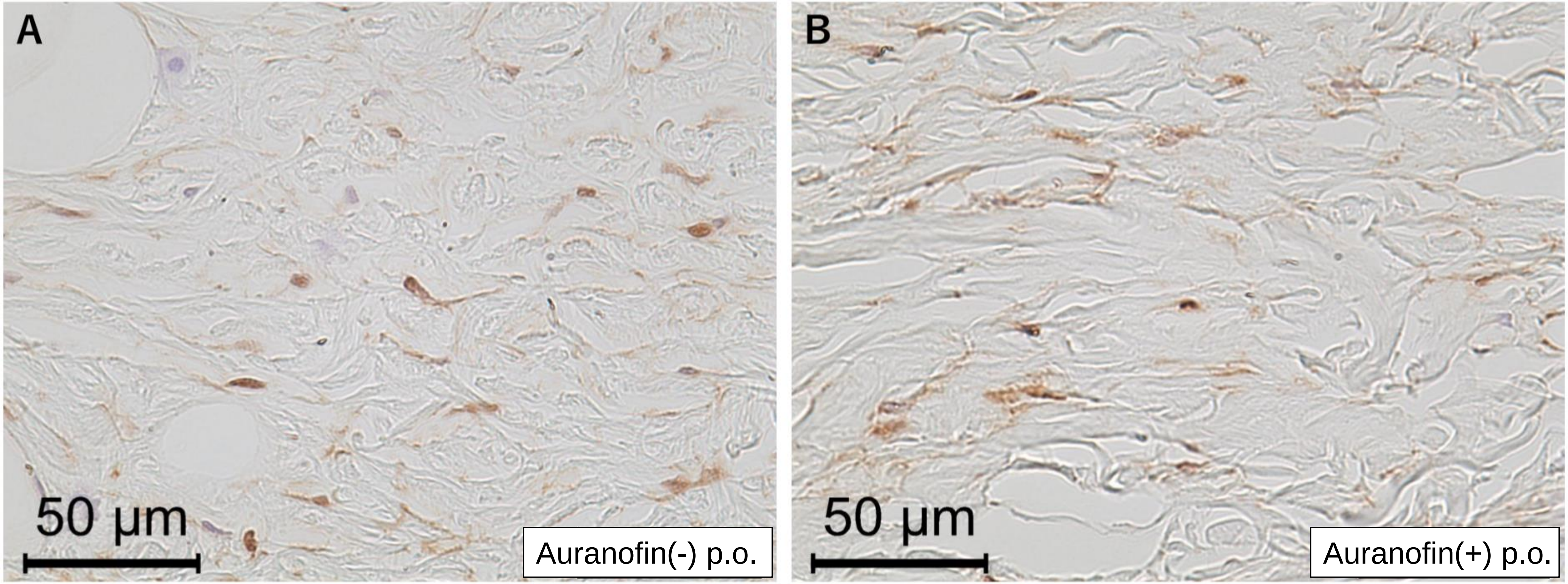

Suppl. Fig. S5. β-catenin immunostaining for tumors in Apc1638N mice.

Developed tumors in Apc1638N mice (6 months of age) with or without treatment of auranofin were subjected to β-catenin immunostaining. Representative images were presented with (A) or without (B) auranofin by oral administration (X 400). p.o. : per OS.

Supplementary Table S1. Changes in protein expression levels of 4 downstream genes of Wnt signaling upon auranofin treatment.

	AXIN2 (94kDa)		CCND1 (36kDa)		MYC (48kDa)		PTGS2 (68kDa)	
	0μM	5μM	0μM	5μM	0μM	5μM	0μM	5μM
Height	321982	15852	114156	10635	76569	16726	133323	16131
Area	3640034	146323	1047397	106971	1221078	220802	3233035	197822

Supplementary Table S2. List of primers used in this study

Gene	Sense	Antisense	Number of base pairs of predicted PCR products
AXIN2	5'-CAACAGATCATCCCATCCAACA-3'	5'-ATTGGGTAGGTGTAAGGAGAC-3'	80 bp
CCND1	5'-ACCAGCTCCTGTGCTGCGAAGTG-3'	5'-GACGGCAGGACCTCCTTCTGCACA-3'	157 bp
MYC	5'-CAGCACCTTCTCATGCATC-3'	5'-AGGATAGTCCTTCCGAGTGG-3'	126 bp
PTGS2	5'-TTGCATTCTTTGCCCAGCAC-3'	5'-TGAAAAGGCGCAGTTTACGC-3'	150 bp
TCF1	5'-CAAGCAGAGTCCAAGGCAGA-3'	5'-AGGATCTGGTTGATGGCAGC-3'	146 bp
TCF1a	5'-AGAACACCCCGATGACGGA-3'	5'-GGCATCATTATGTACCCGGAAT-3'	90 bp
TCF3	5'-GGAGATGAGGGCCAAGGT-3'	5'-TCTGCCTCCTGGACTTGC-3'	258 bp
TCF4	5'-AGAAACGAATCAAAACAGCTCCT-3'	5'-CGGGATTTGTCTCGGAAACTT-3'	84 bp
GAPDH	5'-AGGTCGGAGTCAACGGATTTG-3'	5'-TGTAAACCATGTAGTTGAGGTCA-3'	123 bp
